# Incidental finding of elevated pulmonary arterial pressures during liver transplantation and postoperative pulmonary complications

**DOI:** 10.1186/s12871-022-01839-7

**Published:** 2022-09-21

**Authors:** Alexandre Joosten, François Martin Carrier, Aïmane Menioui, Philippe Van der Linden, Brenton Alexander, Audrey Coilly, Nicolas Golse, Marc-Antoine Allard, Valerio Lucidi, Daniel Azoulay, Salima Naili, Leila Toubal, Maya Moussa, Lydia Karam, Hung Pham, Edita Laukaityte, Youcef Amara, Marc Lanteri-Minet, Didier Samuel, Olivier Sitbon, Marc Humbert, Laurent Savale, Jacques Duranteau

**Affiliations:** 1 Department of Anesthesiology and Intensive Care, Paris-Saclay University, Paul Brousse Hospital, Assistance Publique Hôpitaux de Paris (APHP), 12 Avenue Paul Vaillant Couturier, 94800 Villejuif, France; 2 Department of Anesthesiology and Department of Medicine, Critical Care Division, Centre Hospitalier de L’Université de Montréal, Montréal, Québec Canada; 3 Carrefour de L’innovation Et Santé Des Populations, Centre de Recherche du Centre Hospitalier de L’Université de Montréal, Montréal, Québec Canada; 4 Department of Anesthesiology, Erasme Hospital, Université Libre de Bruxelles, Brussels, Belgium; 5 Department of Anesthesiology, Brugmann Hospital, Université Libre de Bruxelles, Brussels, Belgium; 6 Department of Anesthesiology, University of California San Diego, La Jolla, San Diego, CA USA; 7 Department of Intensive Care, Paris-Saclay University, Paul Brousse Hospital, Assistance Publique Hôpitaux de Paris (APHP), Villejuif, France; 8 Department of Hepatology, Paris-Saclay University, Paul Brousse Hospital, Assistance Publique Hôpitaux de Paris (APHP), Villejuif, France; 9 Department of Hepatobiliary Surgery and Liver Transplantation, Paris-Saclay University, Paul Brousse hospital, Assistance Publique Hôpitaux de Paris (APHP), Villejuif, France; 10 Department of Hepatobiliary Surgery and Liver Transplantation, Erasme Hospital, Brussels, Belgium; 11 Faculty of Medicine, Paris-Saclay University, Le Kremlin-Bicêtre, France; 12 INSERM UMR_S 999, Le Kremlin-Bicêtre, France; 13 Department of Pneumology and Respiratory Intensive Care, Bicêtre Hospital, Le Kremlin-Bicêtre, France

**Keywords:** Liver transplantation, Pulmonary arterial pressure, Postoperative outcomes, Hemodynamic, Liver surgery

## Abstract

**Background:**

In patients with end stage liver disease (ESLD) scheduled for liver transplantation (LT), an intraoperative incidental finding of elevated mean pulmonary arterial pressure (mPAP) may be observed. Its association with patient outcome has not been evaluated. We aimed to estimate the effects of an incidental finding of a mPAP > 20 mmHg during LT on the incidence of pulmonary complications.

**Methods:**

We examined all patients who underwent a LT at Paul-Brousse hospital between January 1,2015 and December 31,2020. Those who received: a LT due to acute liver failure, a combined transplantation, or a retransplantation were excluded, as well as patients for whom known porto-pulmonary hypertension was treated before the LT or patients who underwent a LT for other etiologies than ESLD. Using right sided pulmonary artery catheterization measurements made following anesthesia induction, the study cohort was divided into two groups using a mPAP cutoff of 20 mmHg. The primary outcome was a composite of pulmonary complications. Univariate and multivariable logistic regression analyses were performed to identify variables associated with the primary outcome. Sensitivity analyses of multivariable models were also conducted with other mPAP cutoffs (mPAP ≥ 25 mmHg and ≥ 35 mmHg) and even with mPAP as a continuous variable.

**Results:**

Of 942 patients who underwent a LT, 659 met our inclusion criteria. Among them, 446 patients (67.7%) presented with an elevated mPAP (mPAP of 26.4 ± 5.9 mmHg). When adjusted for confounding factors, an elevated mPAP was not associated with a higher risk of pulmonary complications (adjusted OR: 1.16; 95%CI 0.8–1.7), nor with 90 days-mortality or any other complications. In our sensitivity analyses, we observed a lower prevalence of elevated mPAP when increasing thresholds (235 patients (35.7%) had an elevated mPAP when defined as ≥ 25 mmHg and 41 patients (6.2%) had an elevated mPAP when defined as ≥ 35 mmHg). We did not observe consistent association between a mPAP ≥ 25 mmHg or a mPAP ≥ 35 mmHg and our outcomes.

**Conclusion:**

Incidental finding of elevated mPAP was highly prevalent during LT, but it was not associated with a higher risk of postoperative complications.

**Supplementary Information:**

The online version contains supplementary material available at 10.1186/s12871-022-01839-7.

## Background

Porto-pulmonary hypertension (PoPH) is a well-known complication of portal hypertension, which has been associated with significant morbidity and mortality in patients with end-stage liver disease (ESLD) [1–3]. Its definition relies on the presence of pulmonary artery hypertension that evolves as a consequence of portal hypertension [4]. PoPH is likely caused by an imbalance between vasodilatory and vasoconstrictive mediators causing vasoconstriction, and smooth muscle proliferation and increased pulmonary vascular resistance [5–8]. The diagnosis of PoPH is made by the absence of any cause of pulmonary hypertension other than portal hypertension and by the measurement of a mean pulmonary artery pressure (mPAP) > 20 mmHg, pulmonary vascular resistance > 240 dynes/s/cm^−5^ and pulmonary capillary wedge pressure < 15 mmHg through a right heart catheterization.

Untreated moderate to severe PoPH is usually considered to be a contraindication for liver transplantation (LT) due to its associated high perioperative mortality [9].

At the time of surgery, it is not uncommon to discover elevated mPAP after the placement of the pulmonary artery catheter for patients without a formal preoperative diagnosis of PoPH. This elevated mPAP may be secondary to the “hyperdynamic” hemodynamic state associated with ESLD or, probably more rarely, undiagnosed PoPH. The two situations are very different. The former is not associated with heart failure and may be improved by managing circulating volume overload while the latter is associated with high pulmonary vascular resistance with potential right ventricular failure. Whether or not an elevated mPAP incidentally discovered during LT in the absence of a preoperative diagnosis of PoPH can impact postoperative outcomes has not been investigated.

Postoperative pulmonary complications are frequently reported after a high-risk surgery or LT [10, 11]. Many physiopathological mechanisms have been suggested for these complications, such as respiratory muscle weakness, intraoperative atelectasis and pulmonary oedema [12]. Recent recommendations included an intraoperative restrictive fluid management strategy as part of a preventive bundle for these complications after major surgery, suggesting that pulmonary oedema may play an important role [13, 14]. A high fluid balance has been associated with pulmonary complications in LT. A high mPAP secondary to a hyperdynamic state and circulating volume overload could contribute to pulmonary venous congestion and thus to subclinical pulmonary oedema.

The objectives of this retrospective cohort study were to assess the incidence of intraoperative incidental finding of elevated mPAP in ESLD patients scheduled for LT and its association with different postoperative complications; pulmonary complications being our primary objective. We hypothesized that an incidental high mPAP may be associated with an increased incidence of postoperative pulmonary complications.

## Methods

### Study design and settings

This historical cohort study was conducted at Paul-Brousse hospital (Villejuif, France), a high volume LT center. This study was approved by the Ethics Committee of the French Society of Anesthesia and Resuscitation (IRB# 00,010,254–2020-070) and is reported according to the STROBE guidelines [15]. Written consent was waived by the Ethics Committee.

### Study participants

We identified all patients who underwent a total or partial LT for ESLD between January 01, 2015 and December 31, 2020. We excluded patients who received a combined transplantation (liver-kidney, liver-heart or liver-lung transplants), a retransplantation or a transplantation for acute liver failure, neuropathic amyloidosis or primary liver cancer without known ESLD. We also excluded patients with known PoPH who were treated prior to transplantation and those for whom no baseline mPAP measurement was found on the intraoperative anesthesia health records. All perioperative data were searched using our electronic medical records and anesthesia sheet records.

### Perioperative care

Before surgery, all patients had a preoperative echocardiography and dobutamine stress echocardiogram or myocardial perfusion scintigraphy using positron emission tomography as well as a pulmonary examination including pulmonary function tests. Importantly, the pulmonary artery catheter (swan-ganz catheter) was placed using ultrasound after anesthesia induction Surgical technique was also standardized during the cases. The standard technique used for vena cava reconstruction was the so-called “3-vein piggy-back” technique [7]. In rare cases of caval replacement, a veno-venous bypass was used in case of poor hemodynamic tolerance of caval clamping. More details on the anesthesia and surgical protocol can be found in Supplemental document 1.

### Exposures

Our exposure of interest was the presence of elevated mPAP (equal to or greater than 20 mmHg) at the beginning of surgery in patients with a pulmonary artery catheter inserted and PAP measurements recorded on anesthesia sheet. This threshold is the recognized threshold defining pulmonary hypertension in recent guidelines [4].

### Outcomes

Our primary outcome was a composite outcome of postoperative pulmonary complications which included pneumonia, acute respiratory distress syndrome, acute pulmonary edema, and pleural effusion. This composite outcome is slighy different than other recognized postoperative pulmonary complications composite outcomes [16]. We excluded atelectasis because we hypothetized it might be less relevant for our exposure of interest and added pleural effusion as being more relevant for pulmonary congestive mechanisms. Our secondary outcomes were intraoperative bleeding, need for postoperative renal replacement therapy (in the subgoup of patients who did not require renal replacement therapy in the preoperative period), graft dysfunction, infectious complications (urinary tract infection, sepsis, septic shock, superficial infection, and peritonitis) and 90-day mortality.

All outcomes were prospectively collected by research staff using data from our electronic medical records. Definitions of these complications are reported in Supplemental document 2.

### Covariables

Several preoperative variables were collected to describe the cohort and to adjust for potential confounders. The following variables were considered potential confounders as being potentially associated with a higher mPAP and a higher risk of postoperative complications: age, sex, MELD score, preexisting arterial hypertension (AH), chronic obstructive pulmonary disease (COPD), chronic renal failure (CRF), chronic atrial fibrillation (AF), left ventricular ejection fraction (LVEF) < 50%, and baseline cardiac index.

### Data source and measurement

Some data was available in a database prospectively collected and maintained by our surgical team. We extracted data on missing variables, including our exposure and outcomes, directly from medical charts. MELD was calculated at the inscription on the waiting list and adjusted just before the liver transplant. Outcomes were classified based on reported complications in patients’ charts by treating physicians.

### Statistical analysis

#### Main analysis

We described patients’ characteristics for the full cohort and for each exposure group. We presented categorical variables as frequencies with proportions and continuous variables as means with standard deviations (or medians with first and third quartiles for skewed distributions). We also graphically explored the potential association between baseline mPAP and both the MELD score and the initial cardiac index and estimated LOESS (locally estimated scatterplot smoothing) regressions with 95% confidence intervals. We reported the intensive care unit (ICU) and hospital lengths of stay in a descriptive manner.

We estimated the effect of elevated mPAP on our primary outcome by fitting bivariable and multivariable logistic regression models. We fitted the multivariable model using all potential confounders. For our secondary outcomes, we fitted similar logistic regression models except for blood loss, for which we estimated a log-transformed linear regression model since the distribution of blood loss was right skewed. We explored the statistical interaction between our exposure and the baseline cardiac index in all models. Homoscedasticity and linearity assumptions were explored by an analysis of the residuals for the linear model. The linearity assumption was also explored by fitting a quadratic term for every continuous variable in all models. When the linearity assumption was not met, we fitted polynomial models with quadratic terms to improve the fit of the models. We assessed for the presence of multicollinearity using the Variance Inflation Factor statistic and a cut-off value of 2.5 for all models [17]. We reported odds ratios for the logistic regression models and mean multiplicative factors for the log-transformed linear models as estimates. All estimates were reported with 95% confidence intervals. All statistical analyses were performed using the R software (R collaboration, version 4.0.3).

#### Sensitivity analyses

We conducted sensitivity analyses on the effects of the categorization threshold used for our exposure of interest. Since pulmonary hypertension was up to recently defined as a mPAP above 25 mmHg, we fitted all our multivariable models with such exposure dichotomization. We also conducted a second sensitivity analysis by fitting our multivariable models using a categorization threshold of 35 mmHg for our exposure, which defines moderate pulmonary hypertension and a fourth one with the mPAP as a continuous variable [4]. For the latter, we used restricted cubic splines with 4 knots to explore potential non-linear associations and tested such non-linear associations by conducting either a general linear test or a likelihood ratio test.

## Results

**Fig. 1:**
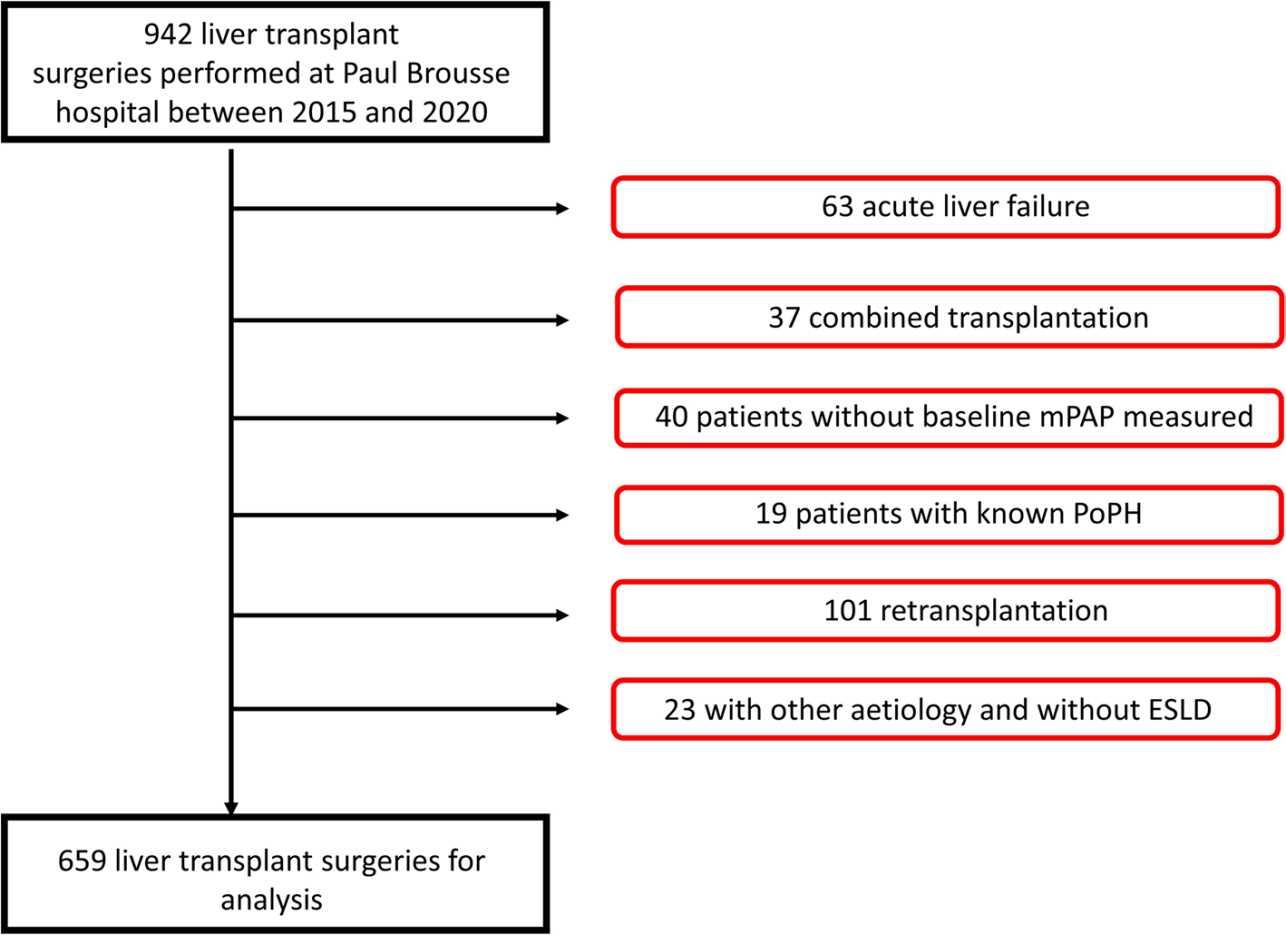
Flow chart

**Fig. 2:**
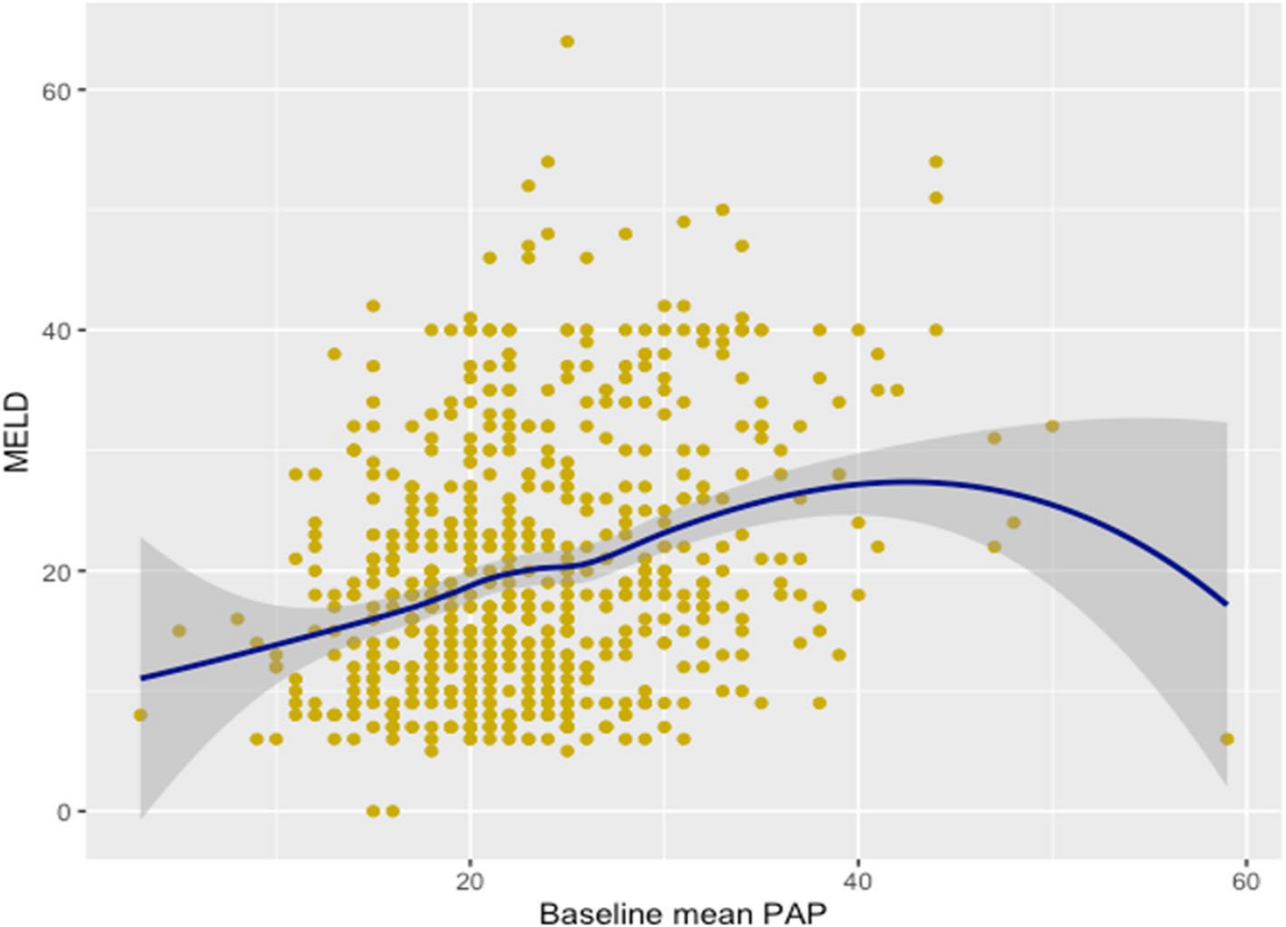
Relation between PAP and MELD. Each observation is represented by a dot. The blue line represents the association between PAP and MELD using a LOESS (LOcally Estimated Scatterplot Smoothing) regression and the grey zone its 95% confidence interval. Where most observations lie, the line suggests that the higher is the PAP, the higher is the MELD. Pearson correlation coefficient with 95% confidence intervals = 0.31 [0.24, 0.38]. This coefficient quantifies the degree to which every point of the diagram falls exactly on a hypothetical straight line and was requested by the reviewers

**Fig. 3:**
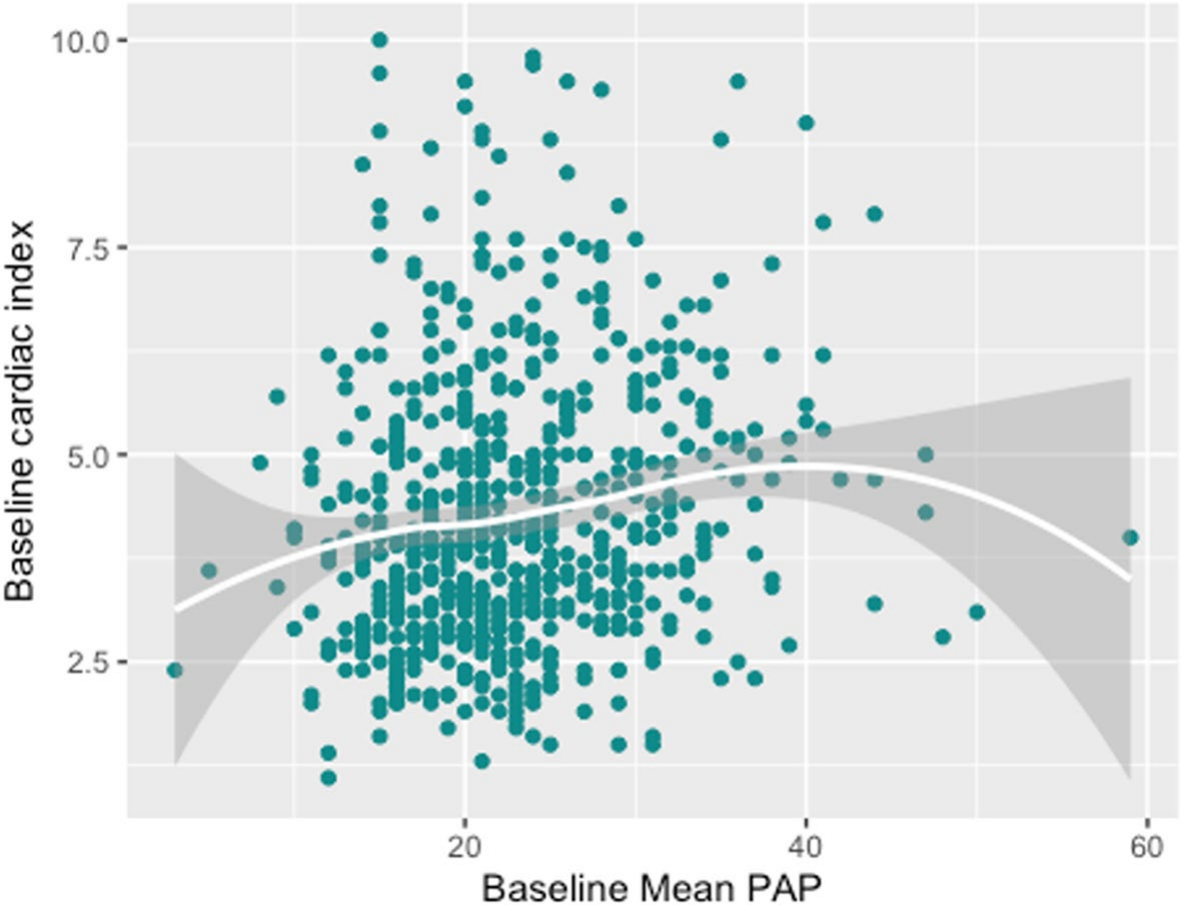
Relation between PAP and cardiac index. Each observation is represented by a dot. The white line represents the association between PAP and cardiac index using a LOESS (LOcally Estimated Scatterplot Smoothing) regression and the grey zone represents its 95% confidence interval. Where most observations lie, the line suggests that the higher is the PAP, the higher is the cardiac index. Pearson correlation coefficient with 95% confidence intervals = 0.16 [0.08, 0.23]. This coefficient quantifies the degree to which every point of the diagram falls exactly on a hypothetical straight line and was requested by the reviewers

Of the 942 LT performed between January 1, 2015 and December 31, 2020, 659 patients met our inclusion criteria. Exclusions are detailed in Fig. 1. Descriptive characteristics of the included patients are reported in Table 1. Relations between baseline mPAP and baseline cardiac index or MELD are reported in Figs. 2 and 3.

**Table 1:** Baseline characteristics

Variables	Full cohort *N* = 659	mPAP ≤ 20 mmHg *N* = 213	mPAP > 20 mmHg *N* = 446
Age (years)	58 [49—65]	57 [44—65]	59 [51—65]
Male sex (%)	476 (72.2)	153 (71.8)	323 (72.4)
MELD score	19.9 (10.7)	16.2 (7.9)	21.7 (11.4)
Arterial hypertension (%)	229 (34.7)	65 (30.5)	164 (36.8)
COPD (%)	90 (13.7)	21 (9.9)	69 (15.5)
Diabetes I (%)	10 (1.5)	4 (1.9)	6 (1.3)
Diabetes II (%)	180 (27.3)	54 (25.4)	126 (28.3)
Asthma (%)	36 (5.5)	15 (7.0)	21 (4.7)
Chronic kidney disease (%)	48 (7.3)	18 (8.5)	30 (6.7)
Preoperative dialysis (%)	18 (2.7)	2 (0.9)	16 (3.6)
LVEJ < 50% (%)	7 (1.1)	1 (0.5)	6 (1.3)
Atrial fibrillation (%)	26 (3.9)	3 (1.4)	23 (5.2)
Initial cardiac index (L/min/m^2^)	4.0 [3.1—5.2]	3.9 [2.9—4.9]	4.4 [3.5—5.6]
mPAP (mmHg)	23.0 (7.1)	15.8 (2.7)	26.4 (5.9)

Data are listed as number and (%) or median and [25–75] percentiles

*MELD* Model for end-stage liver disease, *COPD* Chronic obstructive pulmonary disease, *LVEJ* Left ventricular ejection fraction, *mPAP* mean pulmonary arterial pressure

Among the 659 patients, 446 (67.7%) had an elevated baseline mPAP (mPAP > 20 mmHg). The mean mPAP among those patients were 26.4 (5.9) mmHg. Among the full cohort, 259 patients (39.3%) developed at least one pulmonary complication in the postoperative period (70 patients (32.9%) in the control group and 189 (42.4%) in the elevated mPAP group).

The crude results for the primary and secondary outcomes are reported in Table 2. While a mPAP > 20 mmHg appeared to be significantly associated with an increased risk of pulmonary complications (*unadjusted* OR 1.50; 95% CI 1.07–2.12), this association became closer to a null effect and non-significant when adjusted for confounding factors (*adjusted* OR 1.16; 95% CI 0.80—1.68). Age (*adjusted* OR 1.18; 95% CI 1.03–1.36), MELD score (*adjusted* OR 1.19; 95% CI 1.09–1.29), cardiac index (*adjusted* OR 1.12; 95% CI 1.01–1.25), and the presence of preoperative arterial hypertension (*adjusted* OR 1.83; 95% CI 1.27–2.64) were independently associated with an increased risk of postoperative pulmonary complications (Table 3). We did not observe important association with any of the individual pulmonary complications included in the primary composite outcome (Supplementary Table S1-3).

**Table 2:** Crude results for primary and secondary outcomes

	Full cohort *N* = 659	mPAP ≤ 20 mmHg *N* = 213	mPAP > 20 mmHg *N* = 446
**Primary outcome**			
Postoperative pulmonary complications (%)	259 (39.3)	70 (32.9)	189 (42.4)
**Secondary outcomes**			
Pneumonia (%)	132 (20.0)	35 (16.4)	97 (21.7)
ARDS (%)	42 (6.4)	9 (4.2)	33 (7.4)
Pulmonary edema or pleural effusion (%)	171 (25.9)	50 (23.5)	121 (27.1)
Estimated blood loss (L)	2.5 [1.3—4.1]	2.0 [1.0 -3.6]	2.6 [1.5—4.5]
Dialysis (%)	51 (8.0)	14 (6.6)	37 (8.6)
Graft dysfunction (%)	63 (9.6)	20 (9.4)	43 (9.7)
Infection (any types) (%)	339 (51.5)	101 (47.4)	238 (53.5)
Length of stay in the ICU (hours)	144 [96—244]	120 [96—222]	144 [96—288]
Length of stay in the hospital (days)	20 [15—28]	19 [14—26]	20 [15—29]
Mortality at 90 days (%)	24 (3.6)	7 (3.3)	17 (3.8)

Data are listed as number and (%) or median and [25–75] percentiles

*ARDS* Acute respiratory distress syndrome, *ICU* Intensive care unit, *mPAP* mean pulmonary arterial hypertension

**Table 3:** Pulmonary complications

Variables	Odds ratio and [95% CI]
**Non-adjusted**	
mPAP > 20 mmHg	1.50 [1.07—2.12] *
**Adjusted**	
mPAP > 20 mmHg	1.16 [0.80—1.68]
Age (per 10 years)	1.18 [1.03—1.36] *
Male Sex	0.72 [0.50—1.05]
MELD score (per 5 points)	1.19 [1.09—1.29] *
Cardiac index (L/min/m^2^)	1.12 [1.01—1.25] *
Arterial hypertension	1.83 [1.27—2.64] *
COPD	0.68 [0.41—1.11]
Atrial fibrillation	0.87 [0.35—2.03]
Chronic kidney disease	1.07 [0.57—2.00]
Cardiac insufficiency	0.73 [0.14—3.54]

*mPAP* mean pulmonary arterial pressure, *COPD* Chronic obstructive pulmonary disease, *MELD* Model for end-stage liver disease, *mPAP* mean pulmonary arterial hypertension

^*^p < 0.05

An elevated mPAP was not associated with an increased 90-day mortality (Table 4) and was not associated with any of the other analyzed complications (Supplementary Tables 4 to 7). In our sensitivity analyses, we observed a lower prevalence of elevated mPAP when increasing thresholds (235 patients (35.7%) had an elevated mPAP when defined as ≥ 25 mmHg and 41 patients (6.2%) had an elevated mPAP when defined as ≥ 35 mmHg). We did not observe consistent association between a mPAP ≥ 25 mmHg or a mPAP ≥ 35 mmHg and our outcomes (Supplementary Table S8).

**Table 4:** Mortality at 90 days

Variables	Odds ratio and [95% CI]
**Non-ajusted**	
mPAP > 20 mmHg	1.17 [0.49, 3.06]
**Ajusted**	
mPAP > 20 mmHg	0.99 [0.40, 2.67]
Age (per 10 years)	1.31 [0.91, 2.02]
Male sex	0.88 [0.37, 2.34]
MELD score (per 5 points)	1.16 [0.96, 1.40]
Cardiac index (L/min/m^2^)	0.86 [0.63, 1.14]

*mPAP* mean pulmonary arterial pressure, *MELD* Model for end-stage liver disease

## Discussion

In this single center historical cohort study, we observed a high prevalence (67.7%) of incidental elevated baseline mPAP in patients undergoing a LT for ESLD. We suggested that such incidental finding did not increase risk postoperative pulmonary complications risk. Although an elevated mPAP was significantly associated with an increased risk of postoperative pulmonary complications in the unadjusted analysis, it was no longer associated when important confounding factors were taken into account. Similarly, an elevated mPAP did not appear to be a risk factor for infectious complications, high blood loss, 90-day mortality or any other complications. Such observations did not change when increasing the threshold to define an elevated mPAP (25 or 35 mmHg) while prevalence of an elevated baseline mPAP decreased.

In 2017, DeMartino et al. reviewed hemodynamics of 300 consecutive adult patients undergoing LT and assessed frequency and outcomes of patients with increased mean pulmonary artery pressure (defined as a mPAP ≥ 25 mmHg) at the time of LT. [18] They reported that 39% of recipients had a mPAP ≥ 25 mm Hg and 10.3% had mPAP ≥ 35 mm Hg. They observed that almost all of the cases with high mPAP were caused by a hyperdynamic state with or without hypervolemia and that transplant hospitalization and 1-year posttransplant outcomes were not adversely affected by a high mPAP at time of transplantation. They findings are thus in line with ours and support that most cases of high mPAP are caused by a hyperdynamic state.

While several large retrospective studies have already reported a strong association between pulmonary arterial hypertension and postoperative complications both in major non-cardiac surgery and LT, no study to date has examined the association between an incidental finding of elevated PAP (in previously asymptomatic patients) and postoperative pulmonary complications. A possible explanation is that except in moribund patients or those undergoing either heart or lung transplantation surgery, the pulmonary artery catheter has been progressively replaced by less invasive hemodynamic monitoring approaches during the past two decades. Alternative approaches, such as those utilizing transpulmonary thermodilution techniques (PiCCO ® or VolumeView), have concordantly gained favor among anesthesiology and intensive care teams across the world [19, 20]. However, some LT centers are still commonly utilizing a pulmonary artery catheter, thus making the incidental discovery of pulmonary hypertension a realistic and common occurrence.

A potential clinical impact of this research would be that incidentally discovered intraoperative elevated mPAP does not seem to place LT recipients at increased risk of perioperative morbidity and should not worry us. Nothing specific was done in the postoperative care of the patient. As specified by our pulmonary specialists, such findings seem to be mostly a marker of the severity of the disease and of the hyperdynamic state. Indeed, we did observe in our sample that patients with an incidental finding of elevated mPAP are more likely to be patients with high MELD scores and high cardiac indexes (a “typical” decompensated cirrhotic patients with high MELD and hyperdynamic blood flow). Inversely, preoperative established PoPH is a real risk factor of postoperative complications although recent literature with new treatments is still weak. Our study thus suggests that incidentally discovered elevated mPAP is most probably due to a hyperdynamic state and does not have the negative effects on postoperative outcomes that PoPH may have.

This study has many limitations that must be taken into consideration. First, the design is retrospective and from a single center. Therefore, establishing the existence or lack of a causal relationship with certainty may be limited by exposure, confounder or disease misclassification or measurement errors. Second, since data came from a single institution, external validity may be limited. Third, the pulmonary artery occlusive pressure and pulmonary vascular resistances were not recorded on the anesthesia records, making the exact etiology of the observed high pulmonary pressures difficult to establish. Nevertheless, the relationships between mPAP, MELD scores, and elevated cardiac indexes suggested that most of these patients had decompensating cirrhosis with “passive” hyperdynamic state, as observed in other studies [17].

## Conclusion

An incidental finding of an elevated mPAP was highly prevalent in patients undergoing LT. This incidental finding was not associated with an increased risk of postoperative pulmonary complications or any other postoperative outcomes.

## Supplementary Information

**Additional file 1: Supplemental document 1.** Anesthesia and surgical protocol.

**Additional file 2: Supplemental document 2.** Standardized definition of the primary outcome.

**Additional file 3: Supplementary Table S1-S3.** Analysis on each individual pulmonary complications.

**Additional file 4: Supplementary Tables S4.** Multivariable analysis on blood loss (coefficient multiplicatif).** Supplementary Tables S5.** Dialysis (exclusion of patients with preoperative dialysis). **Supplementary Tables S6.** Graft failure. **Supplementary Tables S7. **Infection.

**Additional file 5: Supplementary Table S8.** Sensibility analysis of multivariable models with other mPAP cutoffs or mPAP as a continuous variable.

## Data Availability

The database is closed and there is no public access. However, permission to access and use the database can be obtained if necessary by request to the corresponding author.

## Abbreviations

PoPH: Porto-pulmonary hypertension
ESLD: End-stage liver disease
LT: Liver transplantation
mPAP: Mean pulmonary arterial pressure
MELD: Model for end-stage liver disease
