# Supplementary material for: Incidental finding of elevated pulmonary arterial pressures during liver transplantation and postoperative pulmonary complications
Source: BMC Anesthesiol. 2022 Sep 21;22:300. doi: 10.1186/s12871-022-01839-7 (PMC9490933; doi:10.1186/s12871-022-01839-7)
Supplement: Supplementary file 1 — Additional file 1: Supplemental document 1. Anesthesia and surgical protocol. [file 12871_2022_1839_MOESM1_ESM.docx]

***Supplemental document 1: Anesthesia and surgical protocol***

Before surgery, all patients had a preoperative echocardiography and dobutamine stress echocardiogram or myocardial perfusion scintigraphy using positron emission tomography as well as a pulmonary examination including pulmonary function tests.

Anesthesia protocol and surgical techniques were standardized in all patients over the study period. Patients arrived in the operating room and were moved onto a heated mattress. The following noninvasive monitors were routinely used: 5-lead electrocardiogram, noninvasive blood pressure, rectal temperature probe, frontal electroencephalogram, Foley catheter, and neuromuscular blockage depth monitoring. Vascular accesses consisted of two large-diameter peripheral venous catheters, a radial arterial catheter, and a right jugular venous catheter placed under ultrasound guidance. The femoral vessels and the left internal jugular vein were left available for a possible veno-venous bypass. A pulmonary artery catheter (swan-ganz catheter) was also placed using ultrasound after anesthesia induction, and hemodynamic management was guided with a calibrated continuous cardiac index monitoring, calibrated venous central oxygen saturation, central venous pressure, and arterial pressure. Rapid infusers and infusion warmers were ready for use prior induction. General anesthesia was induced with sufentanil, propofol or etomidate, and neuromuscular blockade was achieved using succinylcholine (if potassium was in the normal range) and maintained intraoperatively with atracurium. Rapid sequence intubation was performed in all patients. Maintenance of anesthesia was achieved using a sufentanil infusion and inhaled sevoflurane. Fluid administration consisted of a balanced crystalloid infusion (Ringer's lactate) and correction of blood loss with either Ringer's lactate, 4% albumin or packed red blood cells if hemoglobin level was < 8 g/dl.

Surgical technique

In most cases, a total hepatectomy with vena cava preservation was performed associated with a temporary portacaval shunt. The standard technique used for vena cava reconstruction was the so-called “3-vein piggy-back” technique. ^1^ In rare cases of caval replacement, a veno-venous bypass was used in case of poor hemodynamic tolerance of caval clamping.

End-to-end porto-portal anastomosis was preferred in the vast majority of cases, whereas extra-anatomic anastomosis was reserved for more complex situations, as described previously. Hepatic artery anastomosis was usually designed using the junction of the common hepatic artery and gastroduodenal artery, or both recipient’s and graft’s sides. The splenic artery, the recipient's aorta or replaced arteries were considered only as second-intent alternative. Care was taken to avoid grafting an excessively long artery. End-to-end duct-to-duct anastomosis was the preferred technique for biliary reconstruction. A hepaticojejunal stoma with a Roux-en-Y anastomosis was reserved for patients with biliary pathologies or in cases of significant disparities in caliber between the native and grafted bile ducts¨.

**Reference:**

1. Martell M, Coll M, Ezkurdia N, Raurell I, Genescà J. Physiopathology of splanchnic vasodilation in portal hypertension. *World J Hepatol.* 2010;2(6): 208-220
