# Supplementary material for: Incidental finding of elevated pulmonary arterial pressures during liver transplantation and postoperative pulmonary complications
Source: BMC Anesthesiol. 2022 Sep 21;22:300. doi: 10.1186/s12871-022-01839-7 (PMC9490933; doi:10.1186/s12871-022-01839-7)
Supplement: Supplementary file 2 — Additional file 2: Supplemental document 2. Standardized definition of the primary outcome. [file 12871_2022_1839_MOESM2_ESM.docx]

**Supplemental document 2: Standardized definition of the primary outcome**

**Pneumonia**: The presence of new and/or progressive pulmonary infiltrates on chest radiograph plus two or more of the following:

1. Fever ≥ 38.5°C or postoperative hypothermia <36°C

2. Leucocytosis ≥ 10,000 WBC/mm3 or leucopaenia < 4,000 WBC/mm3

3. Purulent sputum and/or

4. New onset or worsening cough or dyspnoea.

**Pleural Effusion**: dyspnea and excessive accumulation of fluid in the pleural space.

**Pulmonary oedema**: respiratory distress or impaired oxygenation AND radiological evidence of pulmonary oedema requiring diuretic therapy

**ARDS**: Acute onset of respiratory failure with hypoxemia as defined by a PaO2/FiO2 ratio ≤ 200 mmHg associated with the development of bilateral opacities or infiltrates on chest radiograph not fully explained by cardiac failure or fluid overload
