# Supplementary material for: Incidental finding of elevated pulmonary arterial pressures during liver transplantation and postoperative pulmonary complications
Source: BMC Anesthesiol. 2022 Sep 21;22:300. doi: 10.1186/s12871-022-01839-7 (PMC9490933; doi:10.1186/s12871-022-01839-7)
Supplement: Supplementary file 3 — Additional file 3: Supplementary Table S1-S3. Analysis on each individual pulmonary complications. [file 12871_2022_1839_MOESM3_ESM.docx]

**Supplementary Table S1-S3. Analysis on each individual pulmonary complications**

**Table S1 : Pneumonia**

| **Variables** | **Odds ratio and [95% CI]** |
| --- | --- |
| **Non-ajusted** | |
| mPAP > 20 mmHg | 1.41 [0.93 - 2.19] |
| **Ajusted** | |
| mPAP > 20 mmHg | 1.01 [0.64 - 1.60] |
| Age (per 10 years) | 1.29 [1.09 - 1.54]* |
| Male sex | 0.91 [0.59 - 1.43] |
| MELD score (per 5 points) | 1.25 [1.14 - 1.38] * |
| Cardiac index (L/min/m^2^) | 1.08 [0.95 -1.23] |

**Table S2 : ARDS**

| **Variables** | **Odds ratio and [95% CI]** |
| --- | --- |
| **Non-ajusted** | |
| mPAP > 20 mmHg | 1.81 [0.89 - 4.09] |
| **Ajusted** | |
| mPAP > 20 mmHg | 1.31 [0.62 - 3.02] |
| Age (per 10 years) | 1.35 [1.01 - 1.86]* |
| Male sex | 0.99 [0.49 - 2.13] |
| MELD score (per 5 points) | 1.22 [1.05 - 1.40]* |
| Cardiac index (L/min/m^2^) | 1.22 [0.91 - 1.36] |

**Table S3: Pulmonary edema and/or pleural effusion**

| **Variables** | **Odds ratio and [95% CI]** |
| --- | --- |
| **Non-ajusted** | |
| mPAP > 20 mmHg | 1.21 [0.83 - 1.78] |
| **Ajusted** | |
| mPAP > 20 mmHg | 1.03 [0.69 - 1.55] |
| Age (per 10 years) | 1.14 [0.99 - 1.32] |
| Male sex | 0.60 [0.41 - 0.89]* |
| MELD score (per 5 points) | 1.08 [0.99 - 1.18] |
| Cardiac index (L/min/m^2^) | 1.12 [0.99 - 1.25] |

*p<0.05

mPAP: mean pulmonary arterial pressure ; MELD : model for end-stage liver disease; ARDS: acute respiratory distress syndrome.
