# Supplementary material for: Incidental finding of elevated pulmonary arterial pressures during liver transplantation and postoperative pulmonary complications
Source: BMC Anesthesiol. 2022 Sep 21;22:300. doi: 10.1186/s12871-022-01839-7 (PMC9490933; doi:10.1186/s12871-022-01839-7)
Supplement: Supplementary file 4 — Additional file 4: Supplementary Tables S4. Multivariable analysis on blood loss (coefficient multiplicatif). Supplementary Tables S5. Dialysis (exclusion of patients with preoperative dialysis). Supplementary Tables S6. Graft failure. Supplementary Tables S7. Infection. [file 12871_2022_1839_MOESM4_ESM.docx]

**Supplementary Tables S4: Multivariable analysis on blood loss (coefficient multiplicatif)**

| **Variables** | **Odds ratio and [95% CI]** |
| --- | --- |
| **Non-ajusté** | |
| mPAP > 20 mmHg | 1.30 [1.3, 1.48]* |
| **Ajusté** | |
| mPAP > 20 mmHg | 1.13 [0.99, 1.29] |
| Age (per 10 years) | 1.01 [0.97, 1.07] |
| Male sex | 1.14 [0.99, 1.30] |
| MELD score (5 points) | 1.28 [1.15, 1.43]* |
| (MELD score (per 5 points))^2^ | 0.98 [0.97, 0.99]* |
| Cardiac index (L/min/m^2^) | 1.25 [1.06, 1.48]* |
| (cardiac index (L/min/m^2^))^2^ | 0.98 [0.97, 0.99]* |
| Arterial hypertension | 1.05 [0.92, 1.20] |
| COPD | 1.07 [0.90, 1.27] |
| Atrial fibrillation | 1.21 [0.89, 1.65] |
| Chronic kidney disease | 0.86 [0.68, 1.08] |
| Cardiac insufficiency | 0.64 [0.36, 1.15] |

*Le coefficient rapporté est un facteur qui multiplie les saignements moyens si la PAPm > 20 mmHg.*

**Supplementary Tables S5. Dialysis (exclusion of patients with preoperative dialysis)**

| **Variables** | **Odds ratio and [95% CI]** |
| --- | --- |
| **Non-ajusted** | |
| mPAP > 20 mmHg | 1.32 [0.72, 2.59] |
| **Ajusted** | |
| mPAP > 20 mmHg | 0.89 [0.45, 1.84] |
| Age (per 10 years) | 0.99 [0.78, 1.28] |
| Male sex | 1.88 [0.91, 4.33] |
| MELD score (5 points) | 0.61 [0.37, 1.02] |
| (MELD (5 points))^2^ | 1.08 [1.03, 1.13]* |
| Cardiac index (L/min/m^2^) | 0.91 [0.74, 1.11] |

**Supplementary Tables S6. Graft failure**

| **Variables** | **Odds ratio and [95% CI]** |
| --- | --- |
| **Non-ajusted** | |
| mPAP > 20 mmHg | 1.03 [0.60 - 1.84] |
| **Ajusted** | |
| mPAP > 20 mmHg | 1.33 [0.74 - 2.46] |
| Age (per 10 years) | 0.72 [0.60 - 0.87]* |
| Male sex | 1.26 [0.70 - 2.39] |
| MELD score (5 points) | 0.89 [0.77 - 1.03] |
| Cardiac index (L/min/m^2^) | 1.00 [0.83 - 1.18] |

**Supplementary Tables S7. Infection**

| **Variables** | **Odds ratio and [95% CI]** |
| --- | --- |
| **Non-ajusté** | |
| mPAP > 20 mmHg | 1.27 [0.92 - 1.77] |
| **Ajusté** | |
| mPAP > 20 mmHg | 1.03 [0.73 - 1.46] |
| Age (per 10 years) | 1.05 [0.93 - 1.20] |
| Male sex | 0.65 [0.45 - 0.93]* |
| MELD score (5 points) | 1.18 [1.08 - 1.28]* |
| Cardiac index (L/min/m^2^) | 1.07 [0.96 - 1.19] |
| Arterial hypertension | 1.12 [0.78 - 1.59] |
| COPD | 1.04 [0.66 - 1.65] |
| Atrial fibrillation | 1.16 [0.51 - 2.68] |
| Chronic kidney disease | 1.10 [0.60 - 2.03] |
| Cardiac insufficiency | 0.51 [0.10 - 2.41] |

*p<0.05

mPAP: mean pulmonary arterial pressure ; COPD : chronic obstructive pulmonary disease

MELD: model for end-stage liver disease
