# Supplementary material for: Incidental finding of elevated pulmonary arterial pressures during liver transplantation and postoperative pulmonary complications
Source: BMC Anesthesiol. 2022 Sep 21;22:300. doi: 10.1186/s12871-022-01839-7 (PMC9490933; doi:10.1186/s12871-022-01839-7)
Supplement: Supplementary file 5 — Additional file 5: Supplementary Table S8. Sensibility analysis of multivariable models with other mPAP cutoffs or mPAP as a continuous variable. [file 12871_2022_1839_MOESM5_ESM.docx]

**Supplementary Table S8: Sensibility analysis of multivariable models with other mPAP cutoffs or mPAP as a continuous variable**

| **Variables** | **mPAP > 20 mmHg** | **mPAP ≥ 25 mmHg** | **mPAP ≥ 35 mmHg** | **mPAP^1^ (continuous)** |
| --- | --- | --- | --- | --- |
| **Pulmonary complications** | 1.01 [0.64 - 1.60] | 1.34 [0.95 - 1.89] | 0.77 [0.38 - 1.53] | 1.03 [0.91, 1.16] |
| **Blood loss** | 1.13 [0.99 - 1.29] | 1.11 [0.98 - 1.27] | 1.11 [0.86 - 1.44] | 1.05 [1.01, 1.09]* |
| **Dialysis** | 0.89 [0.45 - 1.84] | 0.96 [0.49 - 1.82] | 0.61 [0.13 - 2.02] | 0.98 [0.78, 1.22] |
| **Graft dysfunction** | 1.33 [0.74 - 2.46] | 0.88 [0.48 - 1.58] | 0.88 [0.48 - 1.58] | 1.06 [0.87, 1.29] |
| **Infection** | 1.03 [0.73 - 1.46] | 1.22 [0.87 - 1.71] | 1.00 [0.51 - 2.01] | 1.02 [0.91, 1.15] |
| **Mortality at 90 days** | 0.99 [0.40 - 2.67] | 2.48 [1.06 - 6.03]* | 1.19 [0.18 - 4.40] | 1.30 [0.98, 1.68] |

*^1^ by increments of 5 mmHg. None of the outcome had a significant non-linear relationship with the mPAP.*

** statistically significant at alpha = 0.05.*
